# Salivary Metabolic Pathway Alterations in Brazilian E-Cigarette Users †

**DOI:** 10.3390/ijms252111750

**Published:** 2024-11-01

**Authors:** Bruna Fernandes do Carmo Carvalho, Natalia de Carvalho Faria, Kethilyn Chris Sousa Silva, Ellen Greenfield, Mônica Ghislaine Oliveira Alves, Meriellen Dias, Maria Anita Mendes, Mario Pérez-Sayáns, Janete Dias Almeida

**Affiliations:** 1 Instituto de Ciência e Tecnologia, Universidade Estadual Paulista (UNESP), Câmpus São José dos Campos, Av. Eng. Francisco José Longo, 777, São Dimas, São José dos Campos 12245-000, São Paulo, Brazil; brunafernandez@gmail.com (B.F.d.C.C.);; 2 Technology Research Center (NPT), Universidade Mogi das Cruzes, Mogi das Cruzes 08780-911, São Paulo, Brazil; 3 Dempster MS Lab, Department of Chemical Engineering, Polytechnic School, University of São Paulo, São Paulo 05508-040, São Paulo, Brazil; 4 Oral Medicine, Oral Surgery and Implantology Unit (MedOralRes), Faculty of Medicine and Dentistry, Universidade de Santiago de Compostela, 15782 Santiago de Compostela, Spain; 5 ORALRES Group, Instituto de Investigación Sanitaria de Santiago (IDIS), 15782 Santiago de Compostela, Spain; 6 Instituto de los Materiales de Santiago de Compostela (iMATUS), 15782 Santiago de Compostela, Spain

**Keywords:** alcoholic beverages, biomarkers, electronic cigarettes, metabolome, oral health, saliva

## Abstract

In recent years, the use of electronic cigarettes (e-cigs) has increased. However, their long-term effects on oral health and saliva remain poorly understood. Therefore, this study aimed to evaluate the saliva of e-cig users and investigate possible biomarkers. Participants were divided into two groups: the Electronic Cigarette Group (EG)—25 regular and exclusive e-cig users—and Control Group (CG)—25 non-smokers and non-e-cig users, matched in sex and age to the EG. The clinical analysis included the following parameters: age, sex, heart rate, oximetry, capillary blood glucose, carbon monoxide (CO) concentration in exhaled air, and alcohol use disorder identification test (AUDIT). Qualitative and quantitative analyses of saliva included sialometry, viscosity, pH, and cotinine concentrations. Furthermore, the EG and CG salivary metabolomes were compared using gas chromatography coupled with mass spectrometry (GC-MS). Data were analyzed using the Mann–Whitney test. The MetaboAnalyst 6.0 software was used for statistical analysis and biomarker evaluation. The EG showed high means for exhaled CO concentration and AUDIT but lower means for oximetry and salivary viscosity. Furthermore, 10 metabolites (isoleucine, 2-hydroxyglutaric acid, 3-phenyl-lactic acid, linoleic acid, 3-hydroxybutyric acid, 1,6-anhydroglucose, glucuronic acid, valine, stearic acid, and elaidic acid) were abundant in EG but absent in CG. It was concluded that e-cig users had high rates of alcohol consumption and experienced significant impacts on their general health, including increased cotinine and CO concentration in exhaled air, decreased oximetry, and low salivary viscosity. Furthermore, they showed a notable increase in salivary metabolites, especially those related to inflammation, xenobiotic metabolism, and biomass-burning pathways.

## 1. Introduction

Electronic cigarettes (e-cigs) were primarily developed to facilitate smoking cessation through nicotine replacement therapy to help reduce withdrawal symptoms [1]. However, considerable controversy surrounds this topic, with some studies demonstrating that these devices do not aid in smoking cessation but instead significantly increase nicotine dependence in users [2].

The composition of the flavoring used in these devices can vary significantly. Studies have reported metals/metalloids (such as aluminum, antimony, arsenic, cadmium, cobalt, copper, iron, lead, manganese, nickel, tin, and zinc) present in the urine, saliva, serum, and blood samples of e-cig users. Compounds considered cytotoxic and carcinogenic, such as carbonyls, formaldehyde, acetaldehyde, nitrosamines, nitrosonornicotine, and polycyclic aromatic hydrocarbons, have also been reported [3,4,5].

Saliva is a fundamental biofluid that maintains oral balance. Its primary functions include acting as a protective barrier against pathogens, initiating the digestive process, and providing lubrication [6]. In addition, its flow rate and buffering capacity neutralize acids from food or bacterial metabolism, maintain a neutral pH, and balance the processes of dental demineralization and remineralization [7].

Analysis of complex biofluids, such as saliva, urine, blood, and plasma, has gained prominence over the years, as genetic, metabolic, or systemic alterations can precede or indicate the onset of pathologies. Saliva collection is minimally invasive, simple, and cost-effective, making it an attractive technique in omics and promising for identifying potential biomarkers in the oral mucosa and oral microbiota [8,9,10]. The World Health Organization recognizes the carcinogenic potential of certain substances present in e-cigs (NNN and cyanide), which have been identified in the saliva of users [11].

Metabolomics, the study of metabolic profiles in living systems, offers insight into the responses of living systems to pathophysiological stimuli or genetic modifications. These profiles consist of low-molecular-weight metabolites found in living organisms, often resulting from cellular biochemical processes and gene expression [12].

Tumor cells have different metabolic processes than normal cells, which contribute to disease progression. Therefore, enhancing our understanding of cellular metabolism, particularly in the early stages of cellular alteration, is crucial for developing new therapeutic approaches and improving early diagnosis [9]

The literature on salivary assessment and oral changes in e-cig users is scarce. A pilot study published in 2020 [13], which evaluated inflammatory cytokines, and the profile of metabolites present in the saliva of e-cig users, suggests that specific inflammatory pathways linked to periodontal disease may be induced by e-cigs. In addition to high concentrations of cotinine, an increase in the metabolism of arachidonic acid, gangliosides, ceramides, and angiotensin was observed in e-cig users.

Considering the increasing popularity and indiscriminate use of e-cigs by the younger population, as well as the scarcity of the representative literature for the Brazilian population, this study aimed to evaluate possible salivary changes caused by e-cig use.

## 2. Results

### 2.1. Groups Characterization

The groups were mostly composed of men (56%), with an average age of 26–27 years. The groups were paired to justify the absence of any statistical differences. Regarding the epidemiological profile, the groups were similar in terms of self-reported race/skin (mostly white) and educational level (Table 1).

In the physical examination, the EG showed a higher level of exhaled CO (2.12 ± 1.59, *p*-value = 0.015) and a lower oxygen saturation (96.76 ± 1.23, *p*-value = 0.036) than CG (Table 1).

Regarding the qualitative and quantitative saliva analyses, EG presented a lower viscosity (2.04 ± 1.33, *p*-value = 0.048) and a higher cotinine concentration than CG (2.04 ± 1.33, *p*-value = 0.048). No statistical differences were observed in other salivary parameters (Table 1).

### 2.2. Alcoholic Beverage Consumption

The EG had a high alcohol consumption profile. Although the AUDIT score means of both groups remained “low-risk consumption”, the average score of EG was almost twice as high as that of CG (*p*-value = 0.003). When the AUDIT scores were stratified, only 44% of the EG obtained low-risk scores, compared to 92% of the CG (Table 1).

Comparing the mean alcohol doses consumed, 40% of the CG participants consumed one to two doses of alcoholic beverages. In contrast, EG participants consumed significantly more doses than CG participants, with 40% of them consuming at least three to four doses (Table 1).

### 2.3. E-Cig Use Profile

In the profiles of the e-cig users obtained in this study, only 24% were former cigarette smokers (Table 2). Participants in the EG had used e-cigs for at least 2.13 years. In this group, 52% used the devices daily and 60% used them 7–10 times per day. Fruity/sweet flavors were the most consumed flavors, followed by methylating flavors. An average nicotine concentration of 37.2 mg/mL of nicotine was observed, which was considered very high.

An important observation was the simultaneous use of alcoholic beverages and e-cigs (Table 2), with 76% of participants reporting concurrent use. Furthermore, 52% stated that alcohol consumption increased their frequency of e-cig use.

### 2.4. The Salivary Metabolites

**Figure 1:**
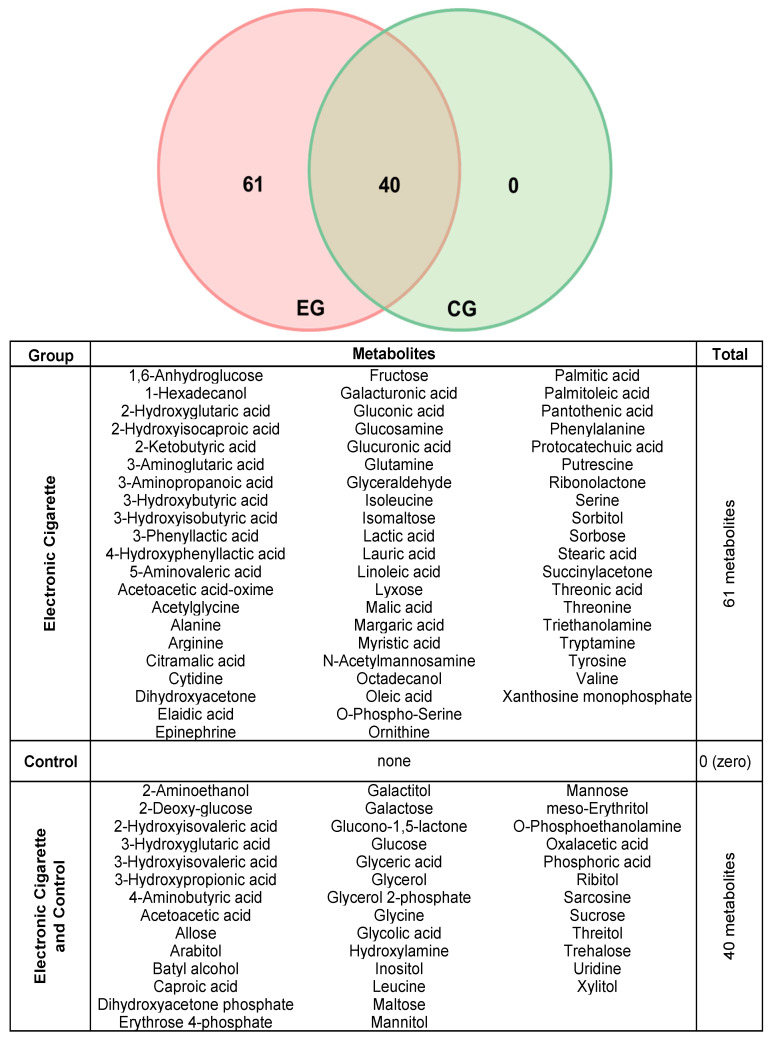
Venn diagram for salivary metabolites probed on E-cig (red) and Control (green) group. The table shows the 61 exclusive metabolites to CG and 40 metabolites shared between EG and CG.

In the raw data table, 342 metabolites were identified, but only those found in at least 70% of the samples were considered for statistical analysis [14]. Therefore, 101 metabolites were included in this study. Of these, 61 metabolites were unique to the EG, while 40 metabolites were shared between the two groups (Figure 1). A total of 3 participants from each group were identified as outliers, resulting in a final sample size of 22 participants per group.

**Figure 2:**
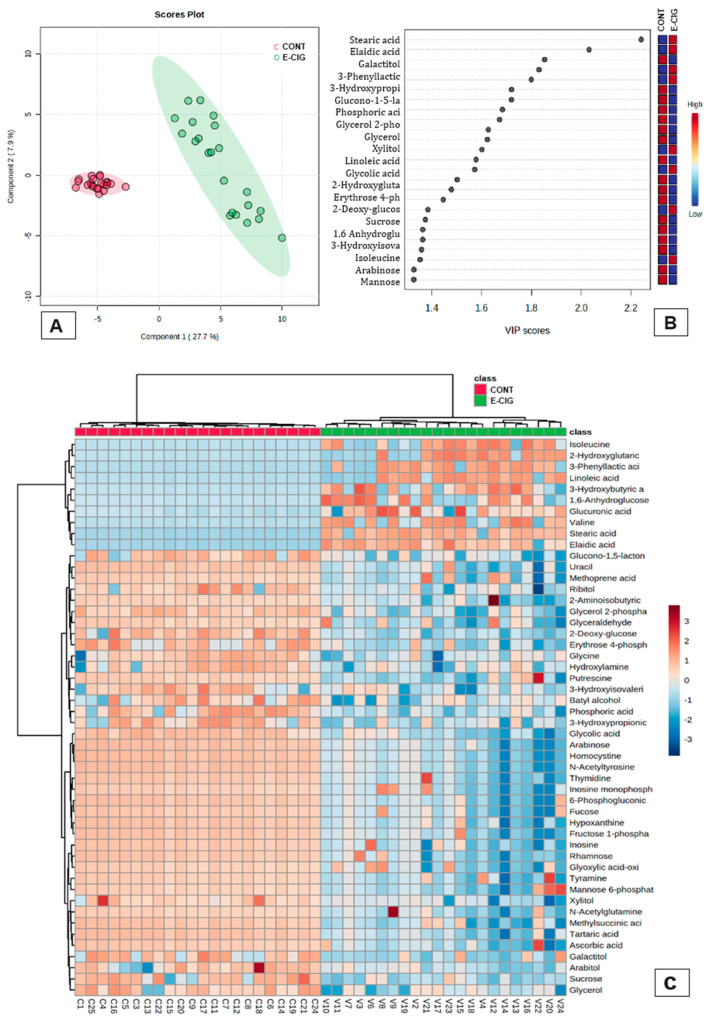
Groups and metabolites evaluation. (**A**) Partial least squares—discriminant analysis—PLS-DA. Red: Control Group. Green: Electronic Cigarette Group. (**B**) VIP score. The high values of concentration are represented in red. (**C**) Heatmap of metabolites to Electronic Cigarette Group (green) and Control Group (Red). The red intensity in the cells indicates higher metabolite concentration in the salivary sample according to the *t*-test (FDR) with q < 0.05.

Figure 2 shows the complete separation of the groups. CG exhibited greater proximity and homogeneity, whereas EG exhibited detachment and heterogeneity.

The clustering of the top 50 metabolites and their concentrations are shown in the heatmap below (Figure 2). CG and EG exhibited statistically different metabolites. The metabolites isoleucine, 2-hydroxyglutaric acid, 3-phenyllactic acid, linoleic acid, 3-hydroxybutyric acid, 1,6-anhydroglucose, glucuronic acid, valine, stearic acid, and elaidic acid were present in EG but absent in CG.

### 2.5. Analysis of Possible Salivary Biomarkers

**Figure 3:**
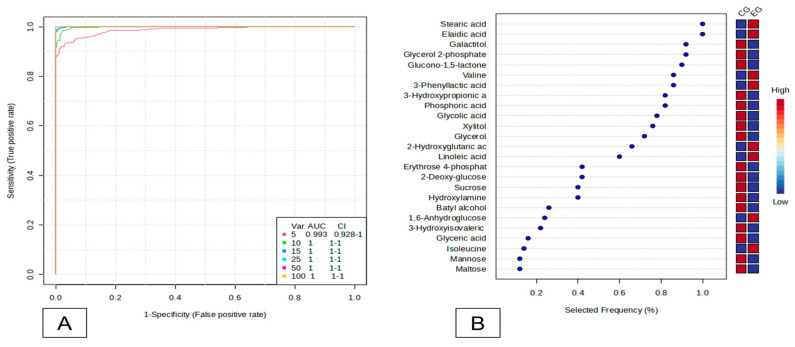
Analysis of salivary biomarkers. (**A**) Multivariate ROC curve. (**B**) Plot of the most important biomarkers based on selected frequency. EG—e-cig group. CG—Control Group.

**Figure 4:**
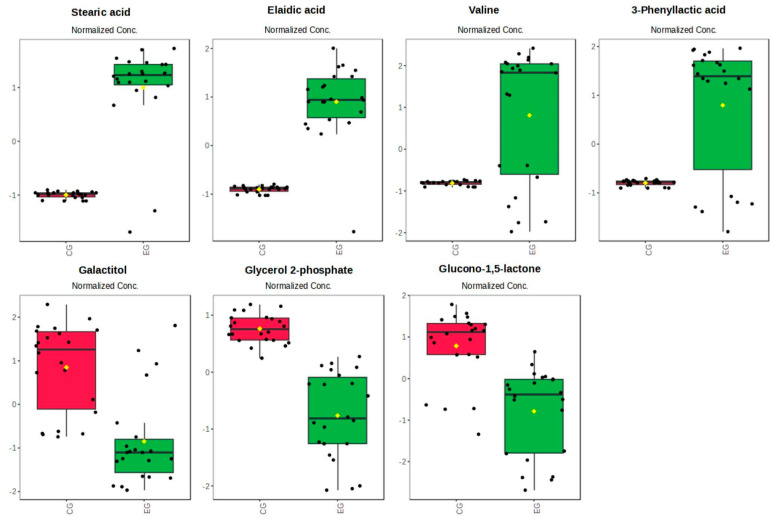
Box plot of significant metabolites. E-cig group (EG—green). Control Group (CG—red).

Using these criteria, seven promising biomarkers were identified (Figure 3). Of these, four were specific and increased in the EG (stearic acid, elaidic acid, valine, and 3-phenyllactic acid), and three were shared between the groups (galactitol, glycerol 2-phosphate, and glucono-1,5-lactone) (Figure 4).

## 3. Discussion

Despite the breadth of information people currently have access to online, the perception that e-cig use does not cause negative health effects seems to persist. E-cig use has been described as the ‘new wave’ of nicotine addiction, and concerns regarding its potential harm to general and oral health have been discussed worldwide [11,15,16]. By analyzing the metabolic profile of saliva from e-cig users without visible clinical changes in the oral mucosa, the findings of the present study may contribute to identifying metabolites and potential biomarkers for the early detection of health changes. To our knowledge, this is the first Brazilian clinical study to evaluate salivary parameters, alcohol consumption, and e-cig usage profile.

Interest in using e-cigs has been growing rapidly, and data revealed that 7 years after the introduction of these devices to the market, approximately 20% of young individuals used the device at least once [17].

The epidemiological profile presented by the EG is similar to the findings in the literature, as the group is predominantly composed of white, highly educated males with a mean age of 27 years [18,19]. Other studies also indicate that young e-cig users, many of whom have no prior cigarette use, often start using these devices through friends or family and later become dual smokers or transition to conventional cigarette use [20,21,22].

### 3.1. E-Cig Effect on General Health

E-cig users appear to be more susceptible to changes in clinical signs, such as arrhythmias, seizures, increased heart rate, and decreased oximetry [23,24,25]. In some cases, pulmonary and cardiac changes can lead to serious conditions, such as pneumonia, COPD (Chronic Obstructive Pulmonary Disease), EVALI (E-cigarette or Vaping Use-Associated Lung Injury), and congestive heart failure [23,24,26]. Other side effects, such as burns and facial trauma, sore throat, dry mouth, shortness of breath, headache, dizziness, and drowsiness, have also been reported [25,27,28].

The findings of the EG clinical evaluation reinforce the reports described above, as the EG showed significant differences in the reduction in oximetry and increase in CO concentration in expired air. Although the CO concentration in expired air can be influenced by the degree of air pollution, these data remain important markers of tobacco consumption [29,30,31]. The association between the decrease in oximetry and the increase in CO leads us to believe that although the clinical damage is still mild, e-cig users already present respiratory changes, which reflect a decrease in the oxygenation capacity of hemoglobin. Long-term damage could not be assessed because this study involved sample collection at a single time point.

### 3.2. Alterations in Salivary Parameters

There are still gaps in the literature regarding the effect of e-cig use on the qualitative–quantitative characteristics of saliva. A reduction in salivary function can lead to several oral health issues, including difficulty in swallowing and speaking, increased risk of dental caries, lesions of the oral mucosa, and periodontal disease [32,33]. Although the viscosity values were the only variable that showed a statistical difference in the present study, we would like to highlight several points that suggest how vaping may influence salivary composition.

The antioxidant capacity of saliva appears to be negatively affected by using e-cigs and can even be compared to the effect observed in cigarette smokers. The defensive capacity of the immune system in the oral cavity is dependent on the salivary antioxidant capacity [34]. Some studies have reported that conventional cigarette smokers may exhibit a lower salivary pH than non-smokers. This reflects that the oral environment of smokers is more acidic and favors the process of dental demineralization [35,36]. In our study, no statistically significant differences were found in salivary pH between the groups.

Although not statistically significant, the salivary flow rate in the EG showed a tendency to decrease compared to the CG. This tendency may be associated with substances, such as propylene glycol and glycerin, found in the flavoring. These substances irritate the upper airways and cause dryness of mucous membranes [37]. Salivary viscosity is directly associated with the amount of mucin present in the saliva. This characteristic plays a crucial role in protecting and hydrating the oral mucosa [34]. Our study indicated a lower viscosity in the EG group than that in the CG group, suggesting that the protein composition of the samples may have undergone changes.

### 3.3. Alcohol and E-Cig Synergism

The e-cig use in conjunction with alcohol abuse is concerning and seems to be very particularly prevalent among the youth, especially in Brazil [38,39].

Tobacco and alcohol consumption are considered the main risk factors for oral cancer. Alcohol acts on the lipid layer of the cell membrane, resulting in gaps in the epithelial layer, which increases the oral mucosa permeability [40]. Furthermore, when metabolized into acetaldehyde, it becomes highly toxic and capable of forming DNA adducts, being mutagenic and carcinogenic [41]. Tobacco, on the other hand, has in its composition, in addition to nitrosamines, several carcinogens such as metabolized N’-nitrosonornicotine (NNN), polycyclic aromatic hydrocarbons, acetaldehyde, and acrolein [42].

In addition to their synergistic effect on oral cancer risk, tobacco and alcohol also impact the oral microbiome, increasing susceptibility to oral diseases and opportunistic infections [43,44].

There have been several discussions on the role of alcohol associated with tobacco (and its derivatives) in the youth. Lozano et al. (2021) [45] reported the relationship between alcohol consumption and e-cig use and the synergistic effect of this relationship. A recent review showed that a reduction in cessation of alcohol consumption decreased the risk of oral and esophageal cancers. The same review revealed some scientific gaps regarding this topic and emphasized the need to address them to develop new strategies for controlling and reducing alcohol use [46]. Accordingly, we observed a higher mean AUDIT score in the EG than in the CG.

Several factors contribute to the association between alcohol and tobacco use among youth, with the most cited explanation being the desire for acceptance within peer groups, particularly among adolescents. This increases their susceptibility to substances, such as tobacco and alcohol [47,48]. Given that the consumption of both substances appears to reinforce each other, they may contribute to the development of substance abuse disorders in adulthood [49,50].

### 3.4. The New Nicotine Use Era

Brazil’s development and economy have a strong historical relationship with the production and consumption of tobacco and alcohol, which have repercussions in the public health sector nowadays [51]. In recent decades, conventional cigarette consumption rates have declined; however, e-cig consumption has increased globally during the same period [52].

In this scenario, the e-cig consumption profile observed in the EG is noteworthy and reflects the global discussion on vaping as a new era of smoking, on par with the consumption of conventional cigarettes. In the EG, we observed a group that used these devices daily, with a high frequency (60% of participants used them more than seven times a day), with a preference for sweet and menthol flavors and high doses of nicotine. Regarding flavorings, the EG was consistent with the findings of Wang et al. (2020) [53], with most participants reporting the use of fruity and/or sweet flavors.

Nicotine is an alkaloid found in its isolated form (free base) or as a salt when combined with an acid (protonated) [54]. In Brazil, the maximum amount of nicotine permitted in each cigarette is 1 mg. However, the nicotine doses observed in this study were higher than those found in a pack of cigarettes [55,56]. These doses were reflected in the high concentration of salivary cotinine in the EG. Cotinine is an important biomarker of smoking, and high doses of cotinine in the saliva, urine, and blood are associated with higher levels of dependence. Mokeem et al. (2018) [57] revealed that cotinine levels in the saliva of e-cig users were similar to those of conventional cigarette users, reinforcing their potential for addiction.

The consumption of free-base nicotine at high concentrations generates a burning sensation and discomfort in the airways, which does not occur with protonated forms [54]. When incorporated into e-cig liquids, it can allow the use of high nicotine concentrations in a more enjoyable manner while ensuring rapid delivery of nicotine to the brain [58].

To understand this new wave of nicotine addiction, we must keep in mind that young people tend to use e-cigs at an earlier age and become increasingly dependent on high doses of nicotine [21,59]. In addition, attractive designs, pleasant tastes, and flavoring smells are also cited as major attractions of e-cigs [60,61].

Individuals under 25 years of age do not yet have a fully developed prefrontal cortex. Nicotine directly affects this area by positively regulating brain receptors, which can lead to issues such as a lack of attention and an increased predisposition to dependence and withdrawal symptoms in the absence of use [62].

Laboratory research indicates that exposure to sweet flavors, compared with traditional menthol and tobacco flavors, increases product appeal and willingness to use, contributing to greater persistence and frequency of use [63,64]. Finally, concerns regarding e-cig use extend beyond the addictive potential of these liquids; as previously mentioned, these devices can also promote several adverse health effects [25].

### 3.5. Salivary Metabolome and Biomarkers

Saliva omics analysis of biomarkers has become increasingly sophisticated and has shown promising results in head and neck cancers, including oral cancer [14,65]. When evaluating the salivary metabolome, it is important to outline the study design because variables found within a group, such as sex, age, tobacco and alcohol consumption, diet, and systemic diseases, can significantly modify the pattern of metabolites observed [66]. Although the present study has some limitations, mainly regarding the number of individuals analyzed, we believe that the grouping and pairing patterns of the groups are a key positive factor. This was also reflected in the quality of the results obtained (as presented in PLS-DA).

When considering smokers, we identified some metabolites that are considered important in literature. While lactate, pyruvate, sucrose, and propylene glycol levels appeared to be higher in smokers, formate levels were lower. These metabolites are relevant because, similar to e-cigs, conventional cigarettes contain sucrose and glucose as flavoring agents and propylene glycol as a humectant [66,67].

In this study, the salivary metabolome profiles of the groups were significantly different. EG presented isoleucine and valine as significant metabolites, and valine was identified as a potential biomarker. In addition to being involved in protein synthesis, branched-chain amino acids (BCAAs), such as leucine, isoleucine, and valine, are precursors of ketone bodies, lipids, and carbon for the synthesis of Krebs cycle intermediates [68].

Increased production of these intermediate components can lead to an imbalance in Krebs cycle reactions, promoting increased histone and DNA methylation and favoring the cellular aging process [69]. Recently, valine was described as an important element in cellular protection against oxidative stress. In addition to increasing the ATP generation rates, it supports the maintenance of oxidative phosphorylation and protects mitochondrial function by preventing oxidative stress-induced damage [70].

Although the protective roles of BCAAs have been widely described, questions remain regarding the impact of their accumulation on the body. The accumulation of BCAAs appears to enhance the inflammatory response and promote senescence, which in turn contributes to the inflammatory environment [71].

The relationship among isoleucine, valine, and smoking or vaping has yet to be established. However, these amino acids appear to be reduced in the saliva of patients with oral squamous cell carcinoma [14,72,73].

E-cig use is associated with increased inflammation, oxidative stress, endothelial dysfunction, and altered lipid homeostasis. In addition, these factors favor the oxidation of fatty acids, such as low-density lipoproteins (LDLs), which are the main factors in atherogenesis [25,74,75]. Gupta et al. (2022) [74] found lower plasma levels of linoleic and arachidonic acids in e-cig users than in conventional cigarette smokers. In the present study, linoleic, elaidic, and stearic acids were identified in EG.

Some unsaturated fatty acids play important roles as precursors of factors in the inflammatory cascade and the composition and function of cell membranes [76]. Linoleic acid is an essential fatty acid that serves as a precursor to arachidonic acid. It cannot be synthesized by the body and is primarily obtained by consuming seeds and vegetable oils [77,78]. The intake of linoleic acid in modest amounts appears to improve overall health. However, excessive intake increases the potential for the formation of oxidized linoleic acid metabolites, which are associated with Alzheimer’s disease, cardiovascular disease, atherosclerosis, liver disease, and some cancers [78,79].

Excess oxidation of elaidic acid, stearic acid, and linoleic acid appears to be associated with the activation of inflammatory pathways, hepatocyte alterations, atherosclerosis, and an increased risk of cardiovascular diseases [80,81,82,83]. In this study, we did not evaluate the diets of the participants. However, we hypothesized that linoleic, elaidic, and stearic acids are related to the composition of the flavors used in e-cigs, which contain vegetable glycerin, propylene glycol, and other vegetable oils in their composition.

Another important salivary metabolite present in the EG is glucuronic acid, which has also been detected in salivary samples from hookah and cigarette users [84]. In human metabolism, the presence of carboxylic acids is associated with the detoxification and metabolism pathways (glucuronidation) of xenobiotic substances, such as carcinogens, environmental toxins, tobacco, and other drugs [85]. The results obtained in this study suggest that e-cig flavoring components, when combined with high alcohol consumption, may activate xenobiotic metabolism pathways.

Increased glucuronic acid levels have been previously reported in the bile of individuals with pancreatic cancer [86]. Animal and cell culture models have shown that the activation of transforming growth factor-beta (TGF-beta) through glucuronic acid dysregulation aggravates the progression and metastasis of hepatocellular carcinoma [87]. Furthermore, enzymatic alterations in glucuronidation have been associated with tumor progression in lung, hepatocellular, and colorectal cancers [88].

Another metabolite present in EG was 1,6-anhydroglucose (or levoglucosan). Levoglucosan is an organic compound formed during the thermal degradation of cellulose and other carbohydrates, such as glucose, fructose, and sucrose [89,90]. Levoglucosan is found in smoke from various types of biomasses, including tobacco [91].

To date, we have not found any studies evaluating the presence of levoglucosan in aerosols or e-cig saliva. Ruprecht et al. (2017) [92] evaluated air from environments exposed to conventional cigarettes, e-cigs, and heat-not-burn tobacco and detected levoglucosan only in conventional cigarettes and heat-not-burn tobacco.

Levoglucosan has been identified in several types of caramel [93]. A study of urinary levoglucosan levels showed that levoglucosan levels were five times higher in individuals who consumed caramel than in those who were exposed to wood smoke. This also suggests a strong dietary influence on urinary levoglucosan levels [94].

As previously mentioned, levoglucosan is associated with direct biomass combustion, such as that occurring in conventional cigarettes [91]. In contrast, e-cigs operate at high temperatures, leading to the vaporization of liquids. Therefore, we suggest that further studies be carried out to investigate whether the presence of levoglucosan in the saliva of e-cig users is related to the high heating temperatures used to vaporize the liquids as well as the presence of sugars, such as those found in caramel.

### 3.6. Limitations of the Study

Despite the growing indiscriminate use of e-cigs, recruiting participants who exclusively use these devices proved challenging. Consequently, the sample size of the present study represents a convenience sample.

Although saliva is used to diagnose some diseases and is a promising biofluid in the field of omics, there are some limitations to be considered [95]:

1. Salivary composition is influenced by time of day, food/beverage intake, collection method, and degree of stimulation.
2. Salivary flow and composition vary across the population and even within individuals, being directly influenced by age, dietary factors, habits, pregnancy/breastfeeding, and hormonal changes.
3. The concentration of salivary markers may not represent the same serum concentrations. Furthermore, oral health conditions or the presence of lesions can alter the concentration of salivary markers.
4. Some medications, oncological treatments, or autoimmune diseases can affect the function of the salivary glands.

It is important to emphasize that in Brazil, the consumption and sale of e-cigs are prohibited by the Brazilian Health Regulatory Agency [96]. We recognize that tobacco and alcohol have distinct effects on saliva and oral cells and that the outcomes may differ when evaluated separately. However, as previously mentioned, it is important to consider the sociocultural aspects of the population evaluated, particularly those who consume these substances concomitantly, as well as the established synergistic factor. Therefore, the inclusion of various types of e-cigs and flavorings, along with individuals who consume both alcohol and e-cigs together, accurately reflects a real-life scenario.

## 4. Material and Methods

### 4.1. Ethical Approval

This study was approved by the Human Research Ethics Committee of the Institute of Science and Technology of São José dos Campos (ICT-UNESP) on 13 November 2020 (CAAE: 36911420.0.0000.0077; approval number: 4.397.780).

### 4.2. Selection of Participants

Two groups were formed for this study:

1. The Electronic Cigarette Group (EG) consisted of 25 regular and exclusive e-cig users for at least 6 months without visible clinical changes in the oral mucosa.
2. The Control Group (CG) comprised 25 non-smokers and non-e-cig users without visible clinical changes in the oral mucosa.

Participants were recruited from São José dos Campos, Mogi das Cruzes, and Jandira between January and August 2022. The individuals who agreed to participate in the study signed a free informed consent form. Individuals over the age of 18 who met the criteria for each specific group were included in the study. Former smokers were eligible for the EG if they had been abstinent for at least 2 years. Exclusion criteria included dual smokers (those using both conventional cigarettes and e-cigs), individuals undergoing treatment for autoimmune diseases, individuals with a history of chronic systemic diseases or on long-term medications (e.g., diabetes, hypertension, hypercholesterolemia, hormone therapy), pregnant and breast-feeding women, and those receiving oncological treatment (surgical, radiotherapy, or chemotherapy).

Extra- and intraoral examinations were conducted to confirm the absence of lesions or oral alterations. Clinical parameters for all participants were established by measuring heart rate, oximetry, and capillary blood glucose were measured. Exhaled carbon monoxide (CO) concentration was assessed using the piCO+ Smokerlyzer^®^ device (Bedfont Scientific Ltd., Harrietsham, UK).

### 4.3. Sample Collection

Before collection, the participants were instructed not to brush their teeth or consume food for 2 h and to abstain from drinking alcohol for 12 h. To reduce oral debris, participants rinsed their mouths with distilled water for 1 min [14].

All samples were collected either in the morning (9:00 a.m. to 11:00 a.m.) or postprandially (2:00 p.m. to 4:00 p.m). Unstimulated saliva was collected for 5 min in a sterile disposable conical tube. Samples were promptly placed on ice, transported to the laboratory, divided into aliquots, and stored at −80 °C until further analysis.

### 4.4. Qualitative and Quantitative Saliva Evaluation

Sialometry (mL/min) was calculated based on the ratio of the amount of saliva obtained in milliliters divided by 5 min. The salivary flow rate was considered very low (hypofunction) at values < 0.1 mL/min, low at 0.1–0.25 mL/min, and normal at values > 0.25 mL/min [97].

Saliva pH was measured using a universal pH indicator paper kit (Merck, Darmstadt, Germany). Each strip was immersed in saliva for 2 s, and the final color was compared to the standard provided by the manufacturer.

Salivary viscosity was evaluated based on saliva thread (in cm). The values of the normal viscosity corresponded to 1.1–1.32 cm, medium viscosity corresponded to 1.32–2.0 cm, and high viscosity corresponded to >2.0 cm [98]

Finally, to determine salivary cotinine levels, a Cotinine Enzyme Immunoassay kit was used (High Quantitative Sensitivity, Salimetrics Inc., State College, PA, USA).

### 4.5. Smoking and Alcoholic Beverage Consumption

The EG participants answered a questionnaire regarding e-cig use, such as age at onset of use, frequency of use, flavoring type, and nicotine.

The alcohol use disorder identification test (AUDIT) was used to assess risk scores related to alcohol consumption in both groups. The AUDIT results are given as the sum of the points; scores ranging from 1 to 7 indicate low-risk consumption, scores 8 to 14 indicate hazardous or harmful alcohol consumption points, and scores of 15 or more indicate moderate-to-severe alcohol use disorder.

### 4.6. Salivary Metabolome Analysis

Initially, saliva samples were thawed at room temperature, and 300 μL were dried in a vacuum centrifuge (Labconco Centrivap Concentrator, Kansas City, MI, USA). Metabolite extraction, derivatization, and salivary metabolome analysis via gas chromatography coupled with mass spectrometry (GC-MS) were performed as described by Alves et al. (2021) [14].

Finally, the spectrometer program generated a raw spreadsheet for the identification and quantification of the metabolite concentrations in each sample.

### 4.7. Statistical Analysis

Epidemiological and clinical data were analyzed using the Mann–Whitney test (*p* values < 0.05) with the PRISM computational program (GraphPad Inc., La Jolla, CA, USA, version 5.03, 2010).

Metabolomic data were analyzed using MetaboAnalyst 6.0 (available at: metaboanalyst.ca/docs/About.xhtml) (accessed on 1 May 2024). The following statistical parameters were used to process the raw data: interquartile range (IQR) of the samples, normalization by median, transformation to the base 10 logarithm, and Pareto scaling. Partial least squares discriminant analysis (PLS-DA), VIP scores, and heat maps were generated.

For biomarker analysis, a multivariate ROC curve was generated using support vector machines (SVMs), PLS-DA, and random forests. Metabolites with an area under the curve greater than 0.8 and a *p*-value of ≥0.05 were considered significant. The Human Metabolome Database (HMDB) and small-molecule pathway database (SMPDB) online platforms were used for metabolite analysis.

## 5. Conclusions

This study revealed significant systemic and salivary alterations in e-cig users. Systemically, there was a decrease in O_2_ saturation and an increase in CO concentration in expired air and cotinine, the principal nicotine metabolite and biomarker, respectively. For saliva, there was a decrease in viscosity and an increase in important metabolites such as isoleucine, 2-hydroxyglutaric acid, 3-phenyllactic acid, linoleic acid, 3-hydroxybutyric acid, 1,6-anhydroglucose, glucuronic acid, valine, stearic acid, and elaidic acid, particularly those related to inflammation, xenobiotic metabolism, and biomass-burning pathways.

## Figures and Tables

**Table 1:** Epidemiological, clinical, and alcohol consumption groups’ profile.

Variables	EG	CG	*p*-Value
Participants	25 (100%)	25 (100%)	NA
Female	11 (44%)	11 (44%)	
Male	14 (56%)	14 (56%)	
Age (in years)	27 ± 7.57	26.80 ± 6.67	0.996
			
Self-reported race/skin color			
White	22 (88%)	20 (80%)	NA
Brown	3 (12%)	4 (16%)	
Black		1 (4%)	
			
Schooling			
High School	2 (8%)		
Ongoing Undergraduate	10 (40%)	13 (52%)	
Undergraduate	9 (36%)	1 (4%)	
Graduate	1 (4%)	11 (44%)	
UD	3 (12%)		
			
Physical examination			
Heart rate	77.72 ± 14.26	81.88 ± 14.53	0.295
Capillary blood glucose (mg/dL)	96.21 ± 17.88	101.90 ± 13.19	0.121
Oximetry (% O_2_)	96.76 ± 1.23	97.56 ± 1.04	0.042 *
CO concentration (ppm)	2.12 ± 1.59	1.48 ± 0.92	0.015 *
			
Saliva parameters			
Sialometry (mL/min)	0.90 ± 0.27	1.24 ± 1.69	0.782
pH	7.12 ± 0.67	7.04 ± 0.57	0.755
Viscosity (cm)	2.04 ± 1.36	2.68 ± 0.9	0.048 *
Cotinine concentration (ng/mL)	46.88 ± 20.01	0.01 ± 0.04	<0.001 *
			
Alcohol consumption			
AUDIT	7.76 ± 4.81	4.04 ± 2.34	0.003 *
Low risk consumption	11 (44%)	23 (92%)	NA
Harmful consumption	8 (32%)	2 (8%)	
Moderate-severe disorder	2 (8%)		
UD	4 (16%)		
			
Average doses consumption			
1 to 2 doses	0 (0%)	10 (40%)	NA
3 to 4 doses	10 (40%)	6 (24%)	
5 to 6 doses	5 (20%)	4 (16%)	
7 to 9 doses	3 (12%)	3 (12%)	
≥10 doses	3 (12%)	0 (0%)	
UD	4 (16%)	2 (8%)	
			
Alcoholic beverages			
Beer	10 (40%)	13 (52%)	NA
Distilled drinks	10 (40%)	5 (20%)	
Wine	1 (4%)	5 (20%)	
UD	4 (16%)	2 (8%)	

The variables are described in the two formats: total number (percentage) or mean ± standard deviation. * denotes *p* ≤ 0.05. EG: Electronic Cigarette Group. CG: Control Group. CO: carbon monoxide. NA: not applicable. UD: unknown data.

**Table 2:** E-cigarette consumption.

Variables	EG
Participants	25 (100%)
E-cig use (in years)	2.13 ± 1.23
Former smoker (industrialized cigarettes)	6 (24%)
Abstinence of industrualized cigarettes (in years)	2.67 ± 0.82
	
E-cig consumption	
1 to 2 days a week	5 (20%)
3 to 4 days a week	7 (28%)
Daily	13 (52%)
	
E-cig frequency of use	
3 to 4 times a day	4 (16%)
5 to 6 times a day	6 (24%)
7 to 10 times a day	3 (12%)
>10 times a day	12 (48%)
	
Vaporization time	
about 1 min	10 (40%)
1 to 2 min	11 (44%)
3 to 5 min	4 (16%)
	
Flavorings	
Flavoring (in mL) per day	8.62 ± 12.65
Nicotine (in mg) per day	37.20 ± 59.95
Nicotine (in mg) per day—UD	5 (20%)
	
Flavoring types	
Fruits and sweet	12 (48%)
Fruits and mint	4 (16%)
Mint and Ice	5 (20%)
UD	4 (16%)
	
Simultaneously e-cig and alcohol use	
Yes	19 (76%)
Sometimes	2 (8%)
No	0 (0%)
UD	4 (16%)
	
Does drinking alcohol increase e-cig use?	
Yes	13 (52%)
Sometimes	4 (16%)
No	4 (16%)
UD	4 (16%)
	
How much alcohol use increases e-cig use?	
1 to 2 times more	6 (24%)
3 to 4 times more	8 (32%)
5 to 6 times more	2 (8%)
I can’t say	5 (20%)
UD	4 (16%)

The variables are described in the two formats: total number (percentage) or mean ± standard deviation. EG: Electronic Cigarette Group. UD: unknown data.

## Data Availability

The data presented in this study are available on request from the corresponding author. The data are not publicly available due to ethical restrictions.
